# Impact of cholesterol on disease progression

**DOI:** 10.7603/s40681-015-0007-8

**Published:** 2015-06-01

**Authors:** Chun-Jung Lin, Cheng-Kuo Lai, Min-Chuan Kao, Lii-Tzu Wu, U-Ging Lo, Li-Chiung Lin, Yu-An Chen, Ho Lin, Jer-Tsong Hsieh, Chih-Ho Lai, Chia-Der Lin

**Affiliations:** 1 Department of Urology, University of Texas Southwestern Medical Center, Texas, Dallas 75235 USA; 2 Graduate Institute of Clinical and Basic Medical Science, School of Medicine, China Medical University, Taichung, 404 Taiwan; 3 Department of Life Sciences, National Chung Hsing University, Taichung, 402 Taiwan; 4 Graduate Institute of Cancer Biology, China Medical University, Taichung, 404 Taiwan; 5 Department of Microbiology and Immunology, Chang Gung University, Taoyuan, 333 Taiwan; 6 Department of Nursing, Asia University, Taichung, 413 Taiwan; 7 Department of Otolaryngology-Head and Neck Surgery, China Medical University and Hospital, Taichung, 404 Taiwan

**Keywords:** Cancer development, Cholesterol, HMG-CoA reductase, Infectious disease, Lipid rafts

## Abstract

Cholesterol-rich microdomains (also called lipid rafts), where platforms for signaling are provided and thought to be associated with microbe-induced pathogenesis and lead to cancer progression. After treatment of cells with cholesterol disrupting or usurping agents, raft-associated proteins and lipids can be dissociated, and this renders the cell structure nonfunctional and therefore mitigates disease severity. This review focuses on the role of cholesterol in disease progression including cancer development and infectious diseases. Understanding the molecular mechanisms of cholesterol in these diseases may provide insight into the development of novel strategies for controlling these diseases in clinical scenarios.

## 1. Metabolism of cholesterol

### 1.1. Biosynthesis of cholesterol

Cholesterol is an extremely important biological molecule as it is a precursor for the synthesis of steroid hormones, bile acids, and vitamin D [1]. The human body manufactures around 1 g of cholesterol each day and approximately 20-25% of total daily cholesterol production occurs in the liver [2]. Synthesis of cholesterol is a series process and starts with acetyl CoA and acetoacetyl-CoA, which are hydrated to form 3-hydroxy-3- methylglutaryl CoA (HMG-CoA). This molecule is subsequently reduced to mevalonate by the enzyme HMG-CoA reductase [3]. This is the regulated, rate-limiting, and irreversible step in cholesterol biosynthesis and is the target of action for statin drugs (HMGCoA reductase competitive inhibitors) [4].

### 1.2. Association of abnormal cholesterol levels with diseases

Both dietary cholesterol and synthesized *de novo* are transported by lipoprotein particles through the circulatory system. The four major types of lipoproteins are chylomicron, very low-density lipoprotein (VLDL), low-density lipoprotein (LDL), and highdensity lipoprotein (HDL). Chylomicrons and VLDL deliver triacylglycerol to cells in the body, whereas LDL delivers cholesterol to cells in the body. Meanwhile, HDL is involved in reverse cholesterol transport. The synthesis and utilization of cholesterol must be tightly regulated in order to prevent over-accumulation and abnormal depositing within the body. There are two manifestations of cholesterol disorders, hyperlipidemia and hypolipidemia. The reasons for cholesterol disorders include dietary issues, genetic disorders, and other diseases [5-7]. For example, due to a genetic disorder caused by a defect on chromosome 19, cholesterol continues to be produced despite there already being an excess of cholesterol in the blood (lack of uptake by LDL receptor), and this may cause familial hypercholesterolemia [8]. In contrast, hypo-cholesterol level may result from liver disease, hypothyroidism, and genetic disorders such as familial hypobetalipoproteinemia and Smith-Lemli-Opitz syndrome (7-dehydrocholesterol reductase deficiency) [9].

**Table 1:** 

Diseases associated with high cholesterol level	References
Atherosclerosis	[11]
Stroke	[12]
Cardiovascular disease (i.e. coronary heart disease and heart attacks)	[13]
Xanthomas (familial hypercholesterolemia)	[14, 15]
Tangier disease (familial HDL deficiency)	[16]
**Diseases associated with low cholesterol level**	
Huntington disease	[17, 8]
Increase in deaths from trauma and hemorrhagic stroke	[19, 20]
Increase risk of neuropsychiatric disorders (i.e. depression , suicide, anxiety, impulsivity, and aggression)	[21-24]

**Table 2:** 

Cancer	Positive related	Negative related
Bladder cancer	[47]	[48]
Breast cancer	[37-40, 49]	[48, 50, 51]
Colon cancer	[44, 45, 52, 53]	–
Female reproductive organ cancer	[46]	[54]
Kidney cancer	[47]	–
Liver cancer	[55]	[53]
Lung cancer	[46, 47]	[53]
Melanoma	[46]	[56, 57]
Non-Hodgkin’s lymphomas	[47]	–
Oral cancer	[41]	[58-60]
Pancreas cancer	[47]	–
Prostate cancer	[61-63]	[47, 48]
Stomach cancer	[47]	[53]

The level of cholesterol in the body being too high or too low may cause varies symptoms, syndromes, or diseases. Excessive cholesterol is associated with several cardiovascular diseases and such levels are easily attained with an unhealthy diet. In fact, it should be noted that it is not essential for cholesterol to be obtained from one’s diet as it is easily synthesized in the body. Whereas, low cholesterol is associated with mental disorders, neuropsychiatric diseases, and mortality in elderly [10]. Some critical diseases related to cholesterol levels are listed in Table 1.

### 1.3. The cholesterol lowering agents

The most important drugs for the treatment of dyslipidemia are statins which have been shown in multiple clinical trials to reduce cardiovascular events and mortality [25]. Statins can inhibit HMG-CoA reductase and design to subsequently inhibit enzyme activity in the liver [26]. Inhibition of cholesterol synthesis further decreases circulating LDL, which reduces levels of cholesterol in the hepatocyte and therefore lead to up-regulated expressions of LDL receptors. Some other drugs have been developed to treat dyslipidemia in specific subsets of patients. For instance, fibrates, which bind to the nuclear receptor PPAR-alpha, can increase HDL levels and decrease triglyceride levels [27]. Fibrates were originally used to address the primary problem of high levels of triglycerides. Another example is niacin (nicotinic acid), which increases HDL levels and decreases triglyceride and LDL levels at high doses (much higher than required for its role as a vitamin) [28, 29]. And there is ezetimibe, which inhibits cholesterol absorption in the small intestine and effectively lowers LDL cholesterol [30].

## 2. Role of cholesterol in cancer progression

### 2.1. Cholesterol and cancer development

Cholesterol is known as a main component of lipid rafts and has been documented to regulate cell membrane proteins, receptor trafficking, signal transduction, as well as influence cell membrane fluidity [31]. Moreover, cholesterol and other lipid-components participate in the production of hormones [32] and energy [33]. However, when large concentrations of cholesterol accumulate in the human body, especially in the organs and blood stream, the risk of various diseases increases (Table 2). Notably, studies have revealed that an increased cholesterol level participates in cancer cell malignancy, and the dysfunction of cholesterol metabolism may also influence cancer progression [34-36]. For example, mevalonate, a cholesterol synthesis precursor, promotes breast cancer cell proliferation *in vivo* and *in vitro* [37, 38]. Additionally, 27- hydroxycholesterol, which is a metabolite from cholesterol, is expressed much higher in the estrogen receptor-positive breast cancer patient site, when compared with both normal breast tissue and a patient’s cancer-free region control [39, 40]. In oral cancer, cholesterol was found to be significantly increased in tumor tissue compared to normal tissue [41]. Moreover, previous studies have reported that elevated cholesterol in the circulatory system promotes Akt signaling, decreases apoptosis activity in LNCap prostate cell line, and enhances tumor aggressiveness in a xenograft animal model [42, 43]. Further, it has been reported that serum cholesterol is a positive factor in colon cancer development [44, 45]. Other cancers, including female reproductive organ cancers, lung cancer, and melanoma are also documented to correlate with high levels of cholesterol [46].

**Table 3:** 

Pathogen	Function	References
*Aggregatibacter actinomycetemcomitan*	CDT holotoxin entry into host cells	[71,72]
*Anaplasma phagocytophilum*	*A. phagocytophilum* infection	[73]
*Campylobacter jejuni*	CDT holotoxin entry into host cells	[74, 75]
*Haemophilus ducreyi*	CDT holotoxin entry into host cells	[76]
*Helicobacter pylori*	CagA translocation and VacA function	[77-80]
HIV	Facilitate HIV infection	[81]
Prion	Promote the conversion of PrP^c^ into the isoform PrP^Sc^	[82]

### 2.2. Reducing cholesterol inhibits cancer progression

In addition to the correlation between cholesterol and cancer progression, disruption of cell membrane lipid rafts or cholesterol components and interference of cholesterol synthesis are considered as treating prospects toward cancer treatment [64, 65]. Therefore, clinical use of cholesterol-controlling medicines has been implied to possess chemoprotective effects [66]. Statins, HMG-CoA reductase inhibitors, are cholesterol-lowering agents [67], and the total consumption of statins has been increasing in recent years [46]. Statins are documented to decrease the proliferation of cancer cells [49, 63], reduce the risk of cancer incidence rate [61], and even influence the mortality rate in cancer patients [68]. However, the findings of statins use in the treatment of cancer have revealed inconsistencies. Some reports have even claimed that the use of statin may increase the risk of cancer [51, 57], or have no correlation in the treatment of cancer [50, 69]. Therefore, the benefits of the cholesterol-controlling aspect in the treatments of lipid rafts-related cancers, animal models, and the details of their underlying mechanisms may need further investigations.

Despite arising number of reports that support the claim that the use of statin significantly reduces the incidence of cancer, not all of the statistical results are consistent with such a claim [48, 70]. Research into cholesterol-related cancer progression and the use of cholesterol-lowering drugs are mostly of the database analysis variety. However, the results may differ according to participant sample selection, sample size, and related confounding factors. Therefore, additional studies with cellular or animal models, long-term vs. short-term statin users follow-up, and even studies consisting of large sample sizes with multiple confounders would help further elucidate this issue.

## 3. Association of cholesterol with pathogen infections

Cholesterol is the most important component of lipid rafts in eukaryotic cells. Lipid rafts are also considered a critical factor in host-pathogen interaction and colonization of hosts by several pathogens including bacteria, viruses, as well as prions. Most of the studies we refer to here describe a few examples of the role of cholesterol in promoting pathogenic infections (Table 3).

### 3.1. Lipid rafts serve as platforms for bacterial pathogens

In order to promote their internalization into host, bacterial pathogens may utilize host cells to enhance their own adherence and survival abilities [83, 84]. Adhesion to host cells by pathogens is the first step in their invasion process and may be associated with lipid rafts. The most commonly described cellular target of intestinal pathogens is *Campylobacter jejuni*, which attach to host epithelial cells via membrane cholesterol [85-87]. In addition, the major virulence factor expressed by C. jejuni is cytolethal distending toxin (CDT) [74], which also can be produced by various common Gram-negative bacteria, including *Aggregatibacter actinomycetemcomitans* [88], *Escherichia coli* [89], *Haemophilus ducreyi* [76], *Helicobacter hepaticus* [90], and *Shigella dysenteriae* [91]. It has been reported that *C. jejuni* CDT-induced pathogenesis of host cells is dependent on membrane cholesterol levels. By using cholesterol-depleting agents such as methylh-β-cyclodextrin (MβCD) which markedly decreased the intoxication of cells [74, 92]. Further evidence of the role of lipid rafts in both *C. jejuni* and *A. actinomycetemcomitans* CDT-induced genotoxicity of host cells have been demonstrated through the cholesterol recognition/interaction amino acid consensus (CRAC) region of the CdtC subunit [71, 75]. These findings indicate that membrane cholesterol provides an essential component for CDT binding to the cell membrane and also serves as a portal for CdtB delivery into host cells for the induction of cell intoxication. Moreover, in this case, the virulence protein cytotoxin-associated gene A (CagA) of *Helicobacter pylori*, is delivered into the target cells by the type IV secretion system [93] and utilizes membrane cholesterol to lead to the activation of pro-inflammatory signaling pathways within gastric cells [75, 78, 94, 95]. Furthermore, a dramatic demonstration of the dissociation of infectivity and pathology is *H. pylori* within encoding glucosyltransferase, which is indispensable for cholesterol glucosylation and promotes *H. pylori*-induced phagocytosis escape and subsequent immune responses [77, 96]. Similar to *C. jejuni* and *H. pylori*, the recent description of the combination of apoE-deficiency and a high cholesterol diet in mice facilitated *Anaplasma phagocytophilum* infection *in vivo* and induced proinflammatory responses [73]. However, not all pathogens require lipid rafts to gain entry into host cells. Recently, it has been shown that cholesterol-mediated invasions and intracellular replication are not required for *Chlamydia trachomatis* and *Salmonella enterica* serovar Typhimurium infection of mice embryonic fibroblasts (MEFs) [97]. Together, these examples illustrate that lipid rafts provide several advantages for bacteria, including virulence factors in modulating internalization and transport of extracellular proteins as well as signaling platforms.

### 3.2. Conversion of prions is associated with lipid rafts

Neurodegenerative disorders caused by prions have been linked to the variant Creutzfeldt-Jakob Disease (vCJD) in humans [98]. The cellular prion protein (PrP^C^) is called a normal cell surface glycoprotein by means of a glycosylphosphatidylinositol (GPI)- anchor. GPI-anchored PrP^C^ is presented in lipid rafts where are microdomains enriched in cholesterol [99]. It is widely known that PrP^C^ is found in membrane cholesterol and plays a crucial role in the development of prion-related diseases by changing its conformation to a pathological isoform (PrP^Sc^) [82]. PrP^Sc^ is an essential part of the prion, causing fatal and transmissible neurodegenerative prion diseases [82]. Several lines of evidence suggest that lipid rafts are highly essential for the transport of PrP^C^ and the toxicity of PrP^Sc^ in neuronal cells [100, 101]. Altogether, these studies indicate the critical role of lipid rafts, which maintain the cell surface localization of GPI-anchor attachment of PrP^C^ and are involved in prion conversion and neurotoxicity.

### 3.3. Lipid rafts facilitate virus infection

Human immunodeficiency virus (HIV) is the retrovirus that is well known to cause acquired immunodeficiency syndrome (AIDS) [102]. Previous clinical evidence indicated that the level of cholesterol may be a potential factor for controlling the spread or fusion of many viruses [103, 104] which are involved in HIV production and infectivity [81]. It has been reported that the negative effector (Nef) protein from HIV can enhance cholesterol uptake and biosynthesis by activating the transcription of the sterol-responsive element binding factor 2 (SREBF-2) and SREBF- 2-regulated genes [105]. In addition, the Nef inhibits the activity of the cellular cholesterol transporter ATP-binding cassette A1 (ABCA1) [106], which in response binds to cholesterol and delivers it to the lipid rafts. Conversely, reduction of cellular cholesterol by ABCA1 activation has been shown to potently inhibit HIV replication [107, 108]. Taken together, these results reveal that HIV requires cholesterol for its egress from and entry into cells.

## 4. Conclusions and perspectives

Cholesterol-enriched microdomains, which provide platforms for signaling, are thought to be associated with the development of various types of cancers. It has also been clear that the role of cholesterol in pathogen-host interactions contributes to further ensure the pathogens’ survival and virulence delivery into host. These findings indicate that an adequate regulation of cholesterol may prevent cancer progression as well as mitigate microbeinduced the pathogenesis of hosts. Fully unveiling the role of cholesterol in diseases’ manifestations may shed light on the possibility to develop a novel approach to the retardation or possible prevention of cancer development and the treatment of infectious diseases.

### Acknowledgments

The authors would like to thank Dr. Ming-Chei Maa and Chang-Mei Lin for their valuable suggestions and editorial assistance. This work was funded by the Ministry of Science and Technology (103-2633-B-039-001 and 103-2991-I-005-507), China Medical University (CMU103-S-15 and CMU103-S-18), and the Tomorrow Medicine Foundation.

### Declaration of interest

The authors declare no conflicts of interest for this work.
